# Understanding Surgical Risk During COVID-19 Pandemic: The Rationale Behind the Decisions

**DOI:** 10.3389/fsurg.2020.00033

**Published:** 2020-05-22

**Authors:** Konstantinos Blouhos, Konstantinos Andreas Boulas, Aikaterini Paraskeva, Alexandros Triantafyllidis, Maria Nathanailidou, Konstantinos Hatzipourganis, Anestis Hatzigeorgiadis

**Affiliations:** Department of General Surgery, General Hospital of Drama, Drama, Greece

**Keywords:** SARS-CoV-2, COVID-19, pandemia, emergency surgery, surgeon, safety

## Introduction

On January 2020, the World Health Organization (WHO) issued a global health alert for a novel coronavirus named severe acute respiratory syndrome coronavirus 2 (SARS-CoV-2) that caused an acute respiratory infection disease (COVID-19) which originated in Wuhan, Hubei Province, China. The COVID-19 pandemic, as declared by the WHO on March 2020, has led to over 770,000 cases worldwide reported at the end of March 2020, spreading on a logarithmic scale in Europe and the USA. As the need for hospitalization among symptomatic cases is ~10% globally, with an increased need for intensive care unit admission and 3% mortality, hospitals have started to intensively reduce elective activities including surgery to prepare for the high numbers of admissions (1).

During SARS outbreaks, health care workers (HCWs) have a significantly increased risk of contracting the SARS-CoV. The risk of acute respiratory infection (ARI) transmission through surgical care services is not fully delineated. This is the reason why WHO guidelines recommend additional infection control measures for surgeons providing services on SARS-CoV-2 suspected patients. Furthermore, the effects of submitting a COVID-19 patient to surgery or general anesthesia on pulmonary functions and postoperative courses are unknown (2). The present article aims to delineate why the surgical patient constitutes a risk factor for SARS-CoV-2 transmission to HCWs while at the same time surgery itself constitutes a risk factor for patient's safety during the COVID-19 pandemia.

## Is There Any Transmission Potential From Asymptomatic SARS-CoV-2 Positive Patients?

It is estimated that a large proportion, almost less than a half of SARS-CoV-2 infected patients, are asymptomatic. Mizumoto et al. reported epidemiological data among the 3,063 passengers of the Diamond Princess cruise ship tested for SARS-CoV-2 with RT-PCR and reported that 634 people tested positive; of the 634 confirmed cases, almost half (51.7%) were reported to be asymptomatic (3). Nishiura et al. reported epidemiological data among 565 Japanese people who had been evacuated from Wuhan, China, and reported that almost a third (30.8%) of them who tested positive for SARS-CoV-2 with RT-PCR was asymptomatic (4).

There are sufficient resources showing that there is a transmission potential from asymptomatic or pre-symptomatic patients. Bai et al. reported a familial cluster of five COVID-19 patients hospitalized with symptoms of ARI that before onset had contact with an asymptomatic family member who remained asymptomatic for the entire duration of surveillance (5). Zou et al. reported that the viral load that was detected in the asymptomatic patient among 18 patients tested positive for SARS-CoV-2 with RT-PCR was similar to that in the 17 symptomatic patients. The authors concluded that asymptomatic or pre-symptomatic patients have a transmission potential, even during the period with modestly detectable viral RNA levels in the oropharynx ~5 days before the onset of symptoms, and suggested that better data are necessary to determine transmission dynamics and inform screening practices (6).

## Why Should All Surgical Patients Be Considered As SARS-CoV-2 Positive Until Proven Otherwise?

It is important to emphasize that surgical patients are a distinct patient category as they can be highly contagious for HCWs under specific conditions during provision of surgical care services. The reason why surgical patients should be treated as highly contagious is that these patients demand close contact and prolonged exposure during surgical care and all are prone to be submitted to aerosol generating procedures, factors that all contribute to ARI transmission.

The majority of surgical patients referred for surgical consultation have no symptoms of ARI. As referred above, approximately half of COVID-19 patients are asymptomatic and have a transmission potential. During the pandemic, it wouldn't be so rare for an asymptomatic or pre-symptomatic COVID-19 patient to have a surgical problem. Consequently, it is rational that all surgical patients, even those without any symptoms of ARI, should be treated as SARS-CoV-2 positive until tested negative.

## Is Surgery A Risk Factor For SARS-CoV-2 Transmission? Routes of Spread

### Transmission Through Infectious Aerosols

The main route of SARS-CoV-2 transmission is inhalation of infectious aerosols. Infectious aerosols consist of large respiratory droplets and droplet nuclei. Large respiratory droplets are >5–10 μm in diameter and are involved in short-range transmission. Droplet nuclei are <5 μm and are responsible for transmission over greater distances. Aerosol generating procedures are defined as procedures that result in the production of droplet nuclei that create the potential for ARI transmission that otherwise may only be transmissible by the droplets.

During perioperative anesthesiological management, intubation, extubation, and related procedures such as manual ventilation are considered as aerosol generating procedures. Tran et al. in a systematic review including five case-control and five retrospective cohort studies evaluated the risk of ARI transmission from aerosol generating procedures and showed that tracheal intubation, non-invasive ventilation, tracheotomy, and manual ventilation before intubation were significantly associated with an increased risk of transmission, whereas endotracheal aspiration, nasogastric tube insertion, nebulizer treatment, high flow O_2_, manipulation of O_2_ mask or BiPAP mask, and chest compressions were not significantly associated with transmission (7). Given the significant concern of airborne transmission, additional infection control measures for surgeons and HCWs providing services on SARS-CoV-2 suspected patients are recommended (8).

### Transmission Through Surgical Smoke: Laparoscopy vs. Laparotomy

As discussed above, the main route of SARS-CoV-2 transmission is inhalation of infectious aerosols. According to angiotensin-converting enzyme 2 (ACE2), expression organs with a high risk for SARS-CoV-2 infection are the lungs followed by kidney, esophagus, bladder, ileum, and heart which also expresses ACE2 (9). Whether transmission is possible through inhalation of aerosols in the surgical smoke produced by energy devices during open and laparoscopic surgery to infected organs is unknown. Furthermore, concerning laparoscopic surgery, there is no evidence to rule out whether leakage of pneumoperitoneum contributes to aerosol transmission.

There is substantial evidence showing the presence of viable viruses in surgical smoke, as activated papillomavirus and HIV. Smoke generated by ultrasonic scalpels has a lower temperature and is more likely to contain more infectious particles than smoke of higher temperatures. Energy devices commonly used in laparoscopy can easily produce large amounts of smoke in the low mobility pneumoperitoneum with significantly higher concentrations than laparotomy. Sudden release of trocar valves, non-airtight exchange of instruments, and small abdominal extraction incisions can expose the surgeon to pneumoperitoneum aerosol exposure (10). Given the significant concern of airborne transmission, conventional instead of laparoscopic surgery is recommended during the COVID-19 pandemia (11).

## Effects of General Anesthesia and Surgery On Postoperative Courses In SARS-CoV-2 Positive Patients

The effects of surgery or general anesthesia on pulmonary functions and postoperative courses in COVID-19 patients are unknown. Aminian et al. in their case series study reported four patients submitted to elective cholecystectomy, hernioplasty, gastric bypass, and hysterectomy and who developed postoperative pulmonary complications in the first few weeks of the COVID-19 outbreak in Tehran, Iran; three out of four patients died due to severe COVID-19 pneumonia and ARDS (12). The above brief clinical report has an apparent importance because of its two key clinical messages: (1) repeated proper documentation is necessary. In their article it is not known whether the four studied patients who submitted to elective surgery were SARS-CoV-2 infected at admission, during hospitalization or after discharge; and (2) the effects of surgery and general anesthesia on COVID-19 progression is unknown. The article clearly highlights the potential fatality that unnecessary surgery can produce during the pandemic.

## Screening For SARS-CoV-2 In All Surgical Patients

Based on the above, screening for SARS-CoV-2 in all surgical patients, in order for unexpected casualties on both the patient and surgeon sides to be avoided, is rational as: (1) surgical patients are highly contagious, (2) surgery is a risk factor for transmission, and (3) the effects of surgery or general anesthesia on pulmonary functions and postoperative courses in COVID-19 patients are unknown.

## Screening-Part 1: History and Physical Examination

At admission to hospital, the surgical patient, who usually has no symptoms of acute febrile respiratory illness plus clinical and epidemiological clues, leaves initial triage and is referred for surgical consultation. At admission to the surgical emergency unit, apart from taking the standard surgical history and making the standard physical examination, surgeons should get familiarized with taking a more thorough epidemiologic history and performing a more thorough physical examination of the respiratory system in search of clues for COVID-19 which should be documented as presented in the module 1 case record form (CRF) provided by the WHO.

## Screening -Part 2: Laboratory, Imaging, and Pathogen Testing

At admission to the surgical emergency unit, apart from the standard laboratory tests, serum lactate (mmol/L), LDH (U/L), CPK (U/L), CRP (mg/L), troponin (ng/mL), ESR (mm/hr), D-dimer (mg/L), ferritin (ng/mL), and IL-6 (pg/mL) levels should be ordered as these markers are essential for proper documentation of patients suspected to have COVID-19 as presented in the module 1 CRF provided by the WHO.

Regarding imaging and pathogen testing, the routine use of chest CT and RT-PCR in all surgical patients for screening and early diagnosis of COVID-19 during the pandemic is rational. As referred above, surgical patients can be highly contagious, asymptomatic SARS-CoV-2 positive patients have a transmission potential, and the effects on disease progression of surgery or general anesthesia in COVID-19 patients are unknown. Therefore, it is of crucial importance that early diagnosis or exclusion of COVID-19 is established if possible before proceeding to surgery for both patient and surgeon safety.

## Screening -Part 3: Repeated Re-Evaluation

Except for the initial detailed documentation as in module 1 WHO CRF, it is of substantial importance that proper documentation is continued during hospitalization and at discharge or death, as in module 2 and 3 WHO CRF. The purpose of detailed documentation is for early diagnosis of a misdiagnosed SARS-CoV-2 positive patient during initial presentation or early diagnosis of an intrahospital transmission.

## In Conclusion

During the COVID-19 pandemic, regardless of the presence of ARI symptoms, all surgical patients should be considered as SARS-CoV-2 suspected: (a) a detailed epidemiologic history and respiratory physical examination should be added to standard history and physical examination; (b) serum lactate, LDH, CPK, CRP, troponin, D-dimer, and ferritin level should be added to standard laboratory testing; (c) chest CT should be added to standard imaging testing; and (d) RT-PCR should be performed in all patients. By gathering all the above data, a rapid risk assessment can be completed for patient triage and decision making based on various pandemic surgery guidelines which share common principles (13). Conclusively, as every surgery entails higher patient and HCW risk, unnecessary exposure to general anesthesia and surgery should be avoided during the COVID-19 pandemic.

## Author Contributions

KBo conceived the idea for the content of this article and drafted the manuscript. AH critically revised the manuscript and provided pivotal feedback on its content. All authors helped shape the final manuscript, gave final approval of the version to be published, and agreed to be accountable for the content of the published article.

## Conflict of Interest

The authors declare that the research was conducted in the absence of any commercial or financial relationships that could be construed as a potential conflict of interest.
